# Thinking about inequalities in physical activity as an emergent feature of complex systems

**DOI:** 10.1186/s12966-024-01659-7

**Published:** 2024-10-29

**Authors:** Sophie Marie Jones, Ana Porroche-Escudero, Katie Shearn, Ruth F. Hunter, Leandro Garcia

**Affiliations:** 1 Centre for Public Health, Queen’s University Belfast, Belfast, UK; 2 Division of Health Research, Lancaster University, Lancaster, UK; 3 Sheffield Hallam University, Sheffield, UK

**Keywords:** Physical activity inequalities, Socioeconomic groups, Complex systems, Systems thinking, Emergence

## Abstract

Inequalities in physical activity are well documented, especially between socioeconomic groups. However, progress on reducing these inequalities is proving challenging. In this commentary, we argue that a complex system perspective is needed, specifically to reorient our thinking of inequalities in physical activity to be emergent features of complex systems. Operationalising this perspective involves acknowledging the multiple dynamic and non-linear interactions which take place between system parts and, over time aggregate to become macro patterns such as physical activity inequalities. We argue that this framing will enhance our understanding of the emergence of inequalities in physical activity and, therefore, provide interventions better suited to the subgroups of the population they are designed to help.

## Introduction

Socioeconomic status (SES) is a complex and multi-dimensional construct broadly referring to an individual’s social standing [33]. Within research, practice and policy, SES is operationalised in many ways including income, education, occupation, and social capital. Additionally, the environments and structures that form society also have SES attributes, which can, directly and indirectly, enhance or hinder people’s access to resources and opportunities across health, social, economic, environmental, and political contexts [9, 20]. The complex layers of SES and the dynamic interactions within the system in which it operates are of interest, particularly concerning the influence of the unequal distribution of individuals engaging in health behaviours [35]. This interest has also been demonstrated in physical activity, with evidence suggesting that physical activity patterns are highly unequal between SES groups [14].

It is well established that individuals who are more educated or have a higher income are more likely to participate in leisure-time physical activity, in comparison to individuals with lower education and income levels, who are more likely to be physically inactive or adopt more utilitarian physical activity (such as occupational or travel physical activity) [2, 49]. Efforts to reduce these inequalities in physical activity levels have been extensive [54]. However, progress is proving to be equally, if not more, challenging than lowering the overall population’s physical inactivity patterns [44].

The lack of progress in reducing inequalities could be explained by two interlinked dominant approaches: (a) the dominant approach towards physical activity, which largely focuses on health-related outcomes (for example, cardiovascular disease or obesity reduction etc.) [27], and overlooks the other economic, social, and environmental benefits of physical activity,and (b) many studies using individualised ‘I-frame’ (individual-frame) [6] and traditional public health approaches. These focus on lifestyle and managing individual behaviour and risk, implying that people have power to control their unhealthy choices, overlooking the fact that “the choices we make are shaped by the choices we have” [37].

Through this commentary, we posit that a complex systems approach – referred to broadly in this paper as a multitude of tools and methods that can help us understand complex systems (definition provided in Table 1) and their parts and the implications these have on observed patterns of behaviour [28] – could help progress our thinking on inequalities in physical activity for two key reasons. Firstly, we agree with Matias and Piggin [27], who argue that there is more to physical activity behaviour than physiological health. They argue the need for a broader view of physical activity to account for interlinked social, emotional, environmental, and political considerations, which shape inequalities in physical activity and contribute to widening health inequalities. Secondly, it is believed here that over emphasising individual risk approaches within behaviour change research and policy are limiting because they shift responsibility to individuals, groups and communities who are already bearing the brunt of social and health inequities rather than acknowledging the broader intertwined contextual, social, political, historical, institutional, and environmental influences that shape individual choices around physical activity [6, 7].

**Table 1:** Glossary of systems related terms

Term	Definition
Complexity	Quality of being complex, that is, of displaying proprieties and behaviours that arise from complex systems. See complex system.
Complex system	Type of system (see system) that arises from the combination of certain conditions (numerosity and diversity of elements; co-evolution between elements, and with its external environment; nested structures; limited or little central coordination; and non-equilibrium – none of which is always present) that lead to one or more of the following properties: self-organisation, emergence, adaptive behaviour, robustness, non-linearity, and path dependency [22]
Emergence, Emergent properties	Macroscopic behaviours, properties and/or functions that arise from the combination of interactions between the system’s factors but are not reducible to the properties of those factors in isolation [34].
Feedback loop	The process of cause-and-effect that exists between two or more factors in a cyclic way (loop) [31]. Feedback loops can either be reinforcing (amplifying the effect of the process over time) or balancing (bringing self-correction to the process over time) [50].
Non-linear, Non-linearity	When a change in the input not necessarily leads to a proportional change of the output.
System	A set of related and interacting factors that form a relatively stable, integrated whole, with macroscopic behaviours, patterns and/or functions that define and are defined by its structure.
Systems approaches	Ways of addressing a problem (frameworks, methods, procedures, techniques, and tools) underpinned by systems thinking principles and concepts, particularly the multiplicity of interacting factors across a system, and the ways in which that system responds and adapts to interventions within it.
Systems thinking	A way of thinking and making sense of the world which is characterised by the application of systems principles and concepts, such as interrelations, self-organisation, feedbacks, adaption and emergence, among others [15].

Based on these arguments, this commentary reasons that a complex systems perspective is needed to reorient how we think about inequalities in physical activity across different SES contexts and groups. Specifically, we discuss physical activity inequalities as an emergent feature of complex systems due to dynamic interactions that produce patterns of behaviour. Throughout the commentary, we use technical terms that may have different interpretations. Their definitions are provided in Table 1.

### Exploring the relationships between socioeconomic groups and physical activity

At the population level, there has been little progress in increasing physical activity over several decades. This is demonstrated by Strain and colleagues [51], who highlight the minimal progress in reducing the overall prevalence of insufficient (i.e., not meeting WHO, national or government recommendations) physical activity levels over the past two decades, with the prevalence of insufficient physical activity in 2022 as 31·3% (95% uncertainty interval 28·6–34·0), an increase from 23·4% (21·1–26·0) in 2000 and 26·4% (24·8–27·9) in 2010. This lack of progress has been particularly extended over recent years, through the COVID-19 pandemic which reduced overall physical activity levels and exacerbated inequalities [21, 36].

Aligned with this lack of progress, there is a limited understanding of what works to successfully enable physical activity across different contexts and for different SES groups [53]. In addition, as highlighted by Salvo and colleagues [46], there is an underrepresentation in this field in lower-and middle-income countries, with less than 20% of peer-reviewed physical activity and health publications being from these countries. As a result, differences in context (including resources available and competing priorities in health, development, and policy) have largely been overlooked [23]. An example of this point can be demonstrated in the reporting of barriers for physical activity. For instance, in higher income contexts, crime and traffic levels have been suggested to act as barriers for individuals to participate in recreational and travel physical activity, however, whilst similar barriers may be present in lower-income contexts, individuals living in these areas (especially those with lower individual levels of income) may have no other option than to utilise travel physical activity for utilitarian purposes [8, 51]. Therefore, the recommendation for physical activity promotion (i.e., intervention design and implementation) in these low-income contexts will be different to the action needed within higher income countries, given the different needs experienced.

Acknowledging this above point also touches on another problem that individuals with lower SES often participate in types of physical activity (travel and occupational) in which they have little or no agency and often in places that are unsafe and not adequately designed to support physical activity [11, 45]. Additionally, whilst the discussion about the health implications (negative and positive) associated with different domains of physical activity are still actively being investigated within the field, previous research has suggested that different domains of physical activity pose different benefits and risks [1, 42]. For instance, the increased risks of occupational physical activity in comparison to leisure-time physical activity, such as increased blood pressure, increased markers for inflammation and therefore, increased risk of cardiovascular diseases [16, 30]. Therefore, individuals with lower SES levels are not only at higher risk for health implications associated with occupational physical activity, but they also miss out on the multidimensional aspects associated with leisure-time physical activity; such as socialization, connectedness with nature, and physical, mental, and affective restoration [14]. As such, these inequalities between domains are also highlighted here as a gap in privilege.

Similarly, recognised sports and exercises are often overpromoted across all SES groups and are based on traditional values and frames [24], underpinned by a host of assumptions about class, race and gender held by the Western world, particularly in high-income countries. We highlight this overpromotion as limiting due to these approaches overlooking “relevant, alternative and already existing ways of living, sports and physical activity” [25], such as dance, whilst also reinforcing social hierarchy by systematically advantaging those with higher SES who have better access, and opportunities for sports participation. Additionally, these assumptions do not engage with the diverse range of thoughts, views, and agencies of the people they are supposed to help [24]. Therefore, we argue that current research and policies may systematically benefit privileged SES groups whilst disadvantaging others despite emphasising the need to move towards more inclusive research and policies better designed to represent diverse groups within the population [26].

Further to the above points, it has also been argued that gaps in understanding inequalities in physical activity across SES groups could be attributed to the overuse of mechanistic, reductionist and linear interventions, commonly employed when designing interventions and their evaluations [5]. In consonance with this argument, challenges of these previous customary approaches are interlinked. First, they focus on finding single causes of inequalities and identifying the mechanisms or factors responsible for predicting and overcoming barriers to being physically active [4]. Second, they tend to focus on social categories (race, gender, class) or individual attributes (i.e., an individual's weight) rather than on the systems of structural disadvantage (racism, poverty). As an unintended consequence, specific social groups may be homogenised and stigmatised since their lack of physical activity is perceived as a consequence of the group’s characteristics, attitudes, and behaviours rather than the result of the intersection of discrimination with inequalities, systems of oppression and specific social categories. Third, as can be inferred from above, context and competing economic, political, historical, and institutional structures make understanding the processes of inequalities difficult due to the multiple factors and layers dynamically interacting in unpredictable and non-linear ways, which causes the observed patterns to be unstable through feedback loops and adaption [17, 29].

For this reason, we argue it is not enough to identify the mechanisms or factors which limit physical activity opportunities; instead, there is a need to understand how the behaviour and the system in which it operates interact with each other to produce the consequent macro patterns we observe [26]. As such, more systems-based research methods (e.g., community-based system dynamics, soft systems methodology, systems-based simulation modelling) are necessary to supplement existing methods to understand the complex relationships that drive various social processes, producing emergent outcomes, such as inequalities in physical activity across SES groups.

Recognising the limitations of solely focusing on individual factors, models such as the Socio-ecological model [43], highlight the many dimensions of physical activity, such as the interpersonal, physical and political environments, which shape physical activity behaviour. This model also identifies that individuals are part of a larger system, and specifically distinguishes the importance of interactions between individuals and environment on health behaviours [43]. Whilst these models and theories show progress by acknowledging the multiple factors, across multiple levels, which influence physical activity, they still lack some, we believe, essential features to fully address the formation of physical activity patterns. For instance, as highlighted by Garcia and colleagues [12], these models tend to overlook the adapting nature of the multiple processes involved in shaping physical activity behaviour, which is pivotal to understand how patterns of behaviour are formed and sustained. Additionally, models such as the socio-ecological model are structured by nested levels (i.e., individual, interpersonal, community and societal levels), forcing boundaries between themselves, which often lacks acknowledgement of the interactions that can occur within or between levels [12]. As such, complex systems approaches are often sought to build on these models and theories to overcome these limitations by recognising the interactions between multiple dimensions which have different level of impact, and consequently allow us to truly understand what is happening in the formation of population patterns (i.e., seeing the bigger picture) [18, 19].

To summarise our points so far, we should aim to broaden current categorisations and conceptualisations of physical activity and move towards a more holistic and inclusive interpretation of physical activity patterns, whereby physical activity is thought of as an essential human need, central to personal, emotional, economic, and social well-being [23, 27]. In doing so, we can elicit a more nuanced understanding of inequalities by honouring diverse contexts, interests and preferences whilst addressing the systematic shift needed across multiple levels and societal structures to reduce inequalities in physical activity across different SES groups.

With this complex systems perspective in mind, in the next section, we highlight our thinking about physical activity inequalities across SES groups as an emergent outcome of the system in which it exists, rather than a distal static factor.

### Thinking about inequalities as an emergent feature of complex systems

We posit that inequalities in physical activity are emergent features of complex systems (see Table 1 for definitions) due to the interplay between the individual and social, economic, political, historical, institutional, and physical environmental factors, which dynamically interact to produce individual and collective physical activity patterns. These factors are self-organising in nature and interact in non-linear ways, which causes each factor to feed back to the system and coevolve due to the dynamic processes involved [19]. Consequently, emergent features, namely in this instance inequalities, arise because of the configuration of the system, (i.e., through the mutually sustained balances between factors and structures within society), which directly and indirectly shape and influence an individual’s opportunities, resources, and capabilities to be physically active, leading to distinctive and imbalanced patterns across different SES groups [17].

**Fig. 1:**
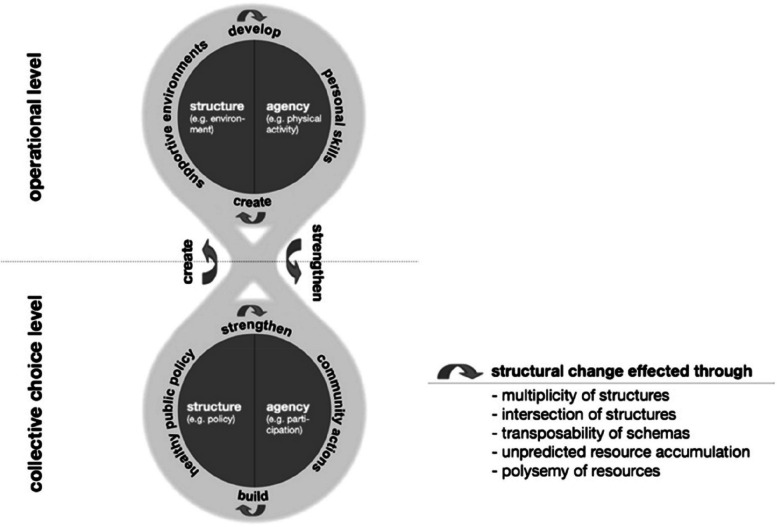
Multi-level interdependence of structure and agency in health promotion [39]

To elaborate further on this idea, we use Rütten and Gelius’s [39] ‘multi-level interdependence of structure and agency in health promotion model’, which centralises the mutually reinforcing constructs of structure and agency, and further demonstrates how system-wide changes can be created and strengthened. A summary of this model is highlighted in Fig. 1.

Specifically, we draw attention to the concept of reinforcement in the model, which highlights the potential of community agency (i.e., participation) to build the structure (of policies), which feed back to strengthen the community’s agency. This can be further demonstrated using an example provided by Rütten and Gelius to highlight the reinforcing relationship between the neighbourhood environment and physical activity levels. At the neighbourhood level, if the area has poor opportunities to be physically active, such as not being conducive to walking or cycling with a high number of traffic collisions, physical activity levels will drop, and thus, the demand for suitable infrastructure may be lower. However, one could argue that improving the infrastructure, in turn, increases physical activity through individuals having more safe and equitable opportunities to be physically active, which then increases the demand for environments conducive to physical activity. This vicious/virtuous cycle does not exist in a vacuum and can be catalysed or constrained by the wider system in which exists, with dynamic interactions with other social, economic, political, historical, and institutional factors. For instance, disfranchised groups may have less institutional spaces to have their views heard and, consequently, policies are less likely to meet their needs, reinforcing inequalities and disfranchisement.

Other researchers have made similar arguments that inequalities in health behaviours (other than physical activity) can be demonstrated as emergent features of complex systems. For instance, when looking at social inequalities in obesity, Matheson [26] highlights obesity as a complex issue driven by non-linear interactions and emergent processes. Specifically, Matheson highlights the ‘downward’ causation’ where emergent patterns impact micro-level interactions, consequently feeding back to the whole system. Figure 1 provides a visual representation of this process. For instance, as Galea and colleagues [10] emphasise, obesity patterns are linked to physical activity patterns; however, obesity could be one contributing aspect of physical activity participation. Therefore, the patterns that emerged over time (obesity) could consequently change the individual’s physical activity behaviour, which could also feedback and exacerbate the patterns observed at the macro-level (inequalities).

Notwithstanding the advantages of utilising the perspective of inequalities as an emergent feature of complex systems, we also acknowledge the difficulty in operationalising complex systems approaches themselves due to the many different interpretations about what exactly constitutes a system, its boundaries, and the consequent different ways these shape physical activity patterns [52].

Therefore, we highlight that in particular, these methods and tools can be used to help researchers, stakeholders and policy makers to work more holistically through collaborating in multisectoral teams [13] whilst also helping to gain a deeper, broader sense of the system through envisioning the current system and the processes and factors which sustain them (i.e., through group model building, or casual loop diagrams as examples) [41]. Additionally, employing a systems approach and specifically considering inequalities as an emergent feature of complex systems can also affect how we design and evaluate interventions [47]. Employing this perspective and consequently using complex systems approaches would mean we are more interested in why the patterns observed occur, (i.e., what the underlying system that shapes and sustains these patterns is and how we may intervene to nudge it to achieve the desired patterns). Approaches such as agent-based models could be used for this purpose [4, 48]. This broadens the scope and timescale for interventions and enables interventions to be better judged in the context in which they are situated.

However, whilst drawing attention to calls for using systems approaches, which have been extensive in the physical activity literature recently [3, 14, 40], robust engagement with the methods is still limited. This is also discussed by Nau et al. [32], who conducted a scoping review of systems approaches for increasing physical activity in populations, and reported that few studies fully engaged with systems concepts, especially with the unique properties of systems approach that distinguish them from social ecological models, that we are more familiar with in physical activity research. Perhaps an explanation for this lack of utilisation is the acknowledgment that research and evaluation is often commissioned and directed towards the performance of the intervention or programme, rather than centring the problem on physical activity inequalities, which has implications for design, implementation, and evaluation [38].

For this reason, it is believed here that we are not yet sure of the full potential of these approaches and how they may be able to work alongside traditional methods to improve our efforts to reduce inequalities in physical activity and improve overall physical activity levels more broadly. Even though the application of systems approaches is still very much in its infancy, we should not be deterred by that and should be encouraged to engage with its use.

Lastly, it is important to emphasise that whilst this commentary is arguing the need for a shift in perspective, we also acknowledge that inequalities, and in particular some determinants of inequalities (e.g., equitable access to suitable infrastructure, social norms etc.) are not easy to modify due to ethical, political and resource constraints [23] in intervention design.

In summary of this commentary, we make three main arguments; the first is to move towards more dynamic and inclusive ways of thinking about physical activity inequalities and related interventions and policies. The second is to acknowledge inequalities as an emergent feature of complex systems, as opposed to a static concept. This brings us to our third argument to utilise complex systems thinking and related approaches to enhance our understanding of inequalities and the dynamic processes involved in producing these inequalities. We believe that it is not until these arguments are realised and applied that we can progress in reducing inequalities in physical activity across SES groups, which, as highlighted in this paper, is not only detrimental from a physical activity viewpoint but also deprives disadvantaged groups of emotional, social, and economic wellbeing.

## Abbreviation

SES: Socioeconomic status
